# Does a social prescribing ‘holistic’ link-worker for older people with complex, multimorbidity improve well-being and frailty and reduce health and social care use and costs? A 12-month before-and-after evaluation

**DOI:** 10.1017/S1463423619000598

**Published:** 2019-09-24

**Authors:** Julian Elston, Felix Gradinger, Sheena Asthana, Caroline Lilley-Woolnough, Sue Wroe, Helen Harman, Richard Byng

**Affiliations:** 1 Researcher-in-Residence, Torbay and South Devon NHS Foundation Trust (TSDFT), Torbay, UK; 2 Research Fellow, Faculty of Medicine and Dentistry, Community and Primary Care Research Group, Plymouth University, UK; 3 Professor, School of Law, Criminology and Government, Faculty of Arts and Humanities, Plymouth University, UK; 4 Project Development Manager, Torbay and South Devon NHS Foundation Trust (TSDFT), Lowes Bridge, Torquay, UK; 5 Chief Officer, Teignbridge CVS, Newton Abbot, Devon, UK; 6 Chief Officer, Age UK Torbay, Torbay, UK; 7 Professor, Community and Primary Care Research Group, Faculty of Health: Medicine, Dentistry and Human Sciences, Plymouth University, UK

**Keywords:** frailty, holistic link-worker, older people, patient activation measure, Researcher-in-Residence, social prescribing

## Abstract

**Aim:**

To evaluate the impact of ‘holistic’ link-workers on service users’ well-being, activation and frailty, and their use of health and social care services and the associated costs.

**Background:**

UK policy is encouraging social prescribing (SP) as a means to improve well-being, self-care and reduce demand on the NHS and social services. However, the evidence to support this policy is generally weak and poorly conceptualised, particularly in relation to frail, older people and patient activation. Torbay and South Devon NHS Foundation Trust, an integrated care organisation, commissioned a Well-being Co-ordinator service to support older adults (≥50 years) with complex health needs (≥2 long-term conditions), as part of its service redesign.

**Methods:**

A before-and-after study measuring health and social well-being, activation and frailty at 12 weeks and primary, community and secondary care service use and cost at 12 months prior and after intervention.

**Findings:**

Most of the 86 participants achieved their goals (85%). On average health and well-being, patient activation and frailty showed a statistically significant improvement in mean score. Mean activity increased for all services (some changes were statistically significant). Forty-four per cent of participants saw a decrease in service use or no change. Thirteen high-cost users (>£5000 change in costs) accounted for 59% of the overall cost increase. This was largely due to significant, rapid escalation in morbidity and frailty. Co-ordinators played a valuable key-worker role, improving the continuity of care, reducing isolation and supporting carers. No entry-level participant characteristic was associated with change in well-being or service use. Larger, better conceptualised, controlled studies are needed to strengthen claims of causality and develop national policy in this area.

## Introduction

In the UK, an ageing population combined with a growing number of people living with long-term medical conditions is increasing demand and cost pressures on the acute, primary and social care services (Wanless *et al.*, 2006; Licchetta and Stelmach, 2016). Large cuts to social service funding (31% real terms between 2011 and 16) (Harris, 2017), stagnating health service budgets due to austerity and concerns about projected levels of funding deficits (Dyson, 2014) have led to policy calls for the redesign of health and social care services. A key demand has been for services to become more integrated to better serve the complex needs of the older, frail population and to be more focused on encouraging supported self-management, as a means to reduce demand on primary and secondary care services, making them more sustainable (Dyson, 2014; NHS England, 2014, 2016a, 2016b).

One innovation that has been consistently advocated for is social prescribing (SP) (Department of Health, 2006; NHS England, 2016a). This is now reflected in the appointment of a national general practitioner (GP) clinical champion (Matthews-King, 2016). It is estimated that 20% of GP appointments have a social element (Parkinson and Buttrick, 2015; Matthews-King, 2016), but GP capacity to address social problems that precipitate and perpetuate ill health are often limited (Popay *et al.*, 2007). SP is a way of connecting patients to practical, community-based support, including access to advice on employment, housing and debt (NHS England, 2016a), as a means of addressing their social, health or economic needs, and promoting well-being and independence. In this way, it is also seen as a way of improving the integration of health and social care, improving patients’ experience (Wilson and Booth, 2015) and reducing demand on primary and acute care service, as well as contributing to other government objectives in relation to employment, volunteering and learning (The King’s Fund, 2018). However, few studies have focused on an older, frail cohort with long-term morbidity.

Furthermore, there is no agreed definition of SP, and models of delivery differ significantly across the UK in relation to the actual activities offered (health, social and economic), and with regard to the level of support given to patients following referral (Moffatt *et al.*, 2017). For example, SP can range from simple signposting to a non-medical local service or a community group by a GP or member of the primary care team, to referral to a link-worker. Link-workers, sometimes based in a GP practice, help determine the person’s needs and connect them to an appropriate local service or resource (Kimberlee, 2015; Husk *et al.*, 2016; Social Prescribing Network, 2016). This role can also vary from ‘light-touch’ (referring people to community assets, typically voluntary transport, befriending, advocacy services) to ‘holistic’(Kimberlee, 2015), a more instrumental, person-centred approach that engages the individual to identify their needs, set well-being goals, and provide practice and emotional support to address these over a period of time, typically three months (Kimberlee, 2015). In contrast to the ‘light-touch’ approach (typically reported in UK studies), which could increase dependency on primary care for addressing social problems and welfare needs (Cawston, 2011), the ‘holistic’ model aims to improve a patient’s self-efficacy and capacity to maintain or improve their health and well-being over the longer term. This is primarily achieved through the relationship with a trained (non-clinical) link-worker. Key elements of this relationship are an open, trusting, non-judgemental, long-term, person-centred relationship, whereby the link-worker acts as a flexible coach, facilitator and patient advocate for support and personal change (Moffatt *et al.*, 2017; Polley *et al.*, 2017b).

In Torbay and South Devon, the Integrated Care Organisation, a provider organisation, commissioned a SP service from the voluntary sector to be integrated into its five locality hubs, alongside primary care, community and social services. Those referred to the service receive an initial strengths-based, guided conversation to determine whether a ‘light-touch’ or ‘holistic’ approach was required. It was assumed that SP would not only improve patients’ mental well-being and ‘activate’ them to better self-manage their health, but would also lead to reduced demand on primary and acute health care and social care services. Studies show that the level of patient activation is a predictor of health care utilisation and cost (Hibbard *et al.*, 2013; Blakemore *et al.*, 2016).

However, recent literature reviews on SP suggest that the evidence base to support these assumptions is small, inconclusive and weak (Bickerdike *et al.*, 2017; Moffatt *et al.*, 2017; Polley *et al.*, 2017a). Only 15 studies were identified with some form of link-worker and these were limited by poor design and reporting (such as small numbers, lack of controls, use of validated measures, short follow-up) and encompassed a mix of delivery models, making them difficult to compare or synthesise (Social Prescribing Network, 2016). Furthermore, many studies failed to disentangle the processes of programme enrolment from the nature of link-worker engagement and adherence to referred activities (Husk *et al.*, 2016) when evaluating effectiveness, making it difficult to attribute causality to these differing components of SP (Husk *et al.*, 2019).

Although studies generally showed positive results, with reports of improvements in health and well-being outcomes, some reductions in use of primary and acute health care and a reduction in costs to the NHS or wider system, many results were not clinically or statistically significant and at a high risk of bias (Moffatt *et al.*, 2017). There was little or no evidence on physical health, patient activation, impact on frailty or use of outpatient, community and social care services or their associated costs.

This study sought to evaluate the impact of a ‘holistic’ link-worker on older patients with multiple long-term conditions across three localities in South Devon. The primary hypothesis was that the intervention would improve health and social well-being, patient activation and frailty levels, and that this would lead to less use of primary, social and acute care services and reduced costs. A secondary objective was to explore what patient characteristics on programme entry were associated with positive outcomes.

## Methods

### Study design

A 12-month before-and-after study followed people receiving the 12-week intensive coaching programme delivered by a Well-being Co-ordinator or ‘holistic’ link-worker in a setting preferred by the person.

### Setting

South Devon is a coastal and moorland area consisting of 140 600 people, mainly resident in 6 market and coastal towns. It has a higher proportion of people over the age of 60 compared to England, and some pockets of deprivation.

The Well-being Coordination service uses 12 Co-ordinators employed by 7 key voluntary sector organisations, embedded in local communities across the area. Co-ordinators are based in a variety of settings, including NHS premises. This study focuses on the South Devon service commissioned by Torbay and South Devon NHS Foundation Trust and managed by Teignbridge CVS, an umbrella voluntary sector organisation. Although the Torbay service (funded by the national lottery and managed by Ageing Well Torbay) operates in a similar way, it was not included as it had its own evaluation.

### Intervention

**Figure 1.**
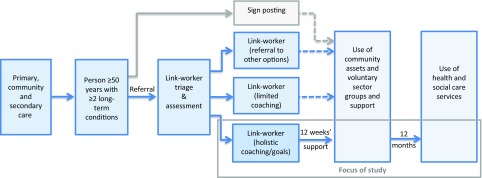
Referral routes and focus of study

The Co-ordinators hold an initial 30–40 min strengths-based, guided conversation with all referrals, mostly in their homes (80%), to determine need and decide whether signposting, a short conversation or a more in-depth ‘holistic’ conversation is required. The latter uses a range of tools over several meetings to enable the person referred to understand what matters to them and set goals for living well. The Co-ordinator then works with the individual for up to 12 weeks to enable them to take action to achieve their goals. This includes resilience-focused coaching and practical support and advocacy to navigate and access local health, social and economic services. This study focuses just on those participants receiving the more intensive ‘holistic’ intervention (see Figure 1).

All Co-ordinators are non-health care staff (although some previously worked in the health service) and all received training in goal setting, use of tools and outcome measures, and in how to engage with users in a strengths-based way, co-produce a plan and manage risk. Key aspects of the role included: listening skills, emotional support, advice and practical assistance and coaching.

### Participants

Participants were individuals aged 50 years or over with two or more long-term conditions, considered as likely to benefit from a social intervention. Referrals were received mainly from statutory services – GPs, community and social care staff in multidisciplinary meetings, hospital discharge staff (acute and community) and housing staff – as well as local voluntary and community organisations. Figure 1 shows the referral routes and the focus of the study.

### Variables

Data on age, sex, referral route were recorded on referral forms and health outcomes collected at either the first or second meeting with the Co-ordinator and after 12 weeks support (or the point of exit). The following validated health outcome measures were used: Well-being Star™ (seven domains, each scoring 1–10), Patient Activation Measure (PAM)® (13 items, scoring 0–100, or levels 1–4) and Warwick-Edinburgh Mental Health and Well-being Scale (WEMWBS) (14 Likert questions, each scoring 1–5) (Tennant *et al.*, 2007) and the Rockwood Clinical Frailty Scale (RCFS) [scoring 1 (very fit) to 10 (terminally ill), with frailty ≥5] (Rockwood *et al.*, 2005).

Referrals from GP practices, social care staff and voluntary sector organisations were categorised as Community referrals, and those from acute services or intermediate care multidisciplinary teams (MDT) as Complex referrals.

### Data sources/measurement

Data on the use of health and social care services were collated from local IT systems 12 months prior to and after the date of each referral. It included the following services: accident & emergency (A&E) and minor injury units (MIU), in-patient, outpatient, community service (ie, occupational therapy, physiotherapists and nursing) and social service contacts and length of stay (in-patients only) and GP contacts. Contacts outside the clinical commissioning group (CCG), boundary were also included.

### Cost data

The cost analysis was based on health and community service attendance 12 months before and after the intervention. It used the Trust’s attendance costs submitted to the National Tariff Payment System for 2016–17, rounded to the nearest £10, and where this involved an admission, was multiplied by length of stay (days). Costs included overheads [ie, building and maintenance (including equipment), administration and back office staffing costs], and nurse and doctor time (except in-patient costs) (Monitor and NHS England, 2016). Diagnostics costs were not included. Local estimates were: A&E (£80), in-patient care (£300 per day), outpatient (£100) and community services (£70). Social care costs were based on actual expenditure, using figures from contract arrangements with social services. The per person cost of the well-being coordination (WBC), service was not included in calculations.

### Bias

Data were collected by the same link-worker and returned to the administrator. The NHS number was used as the key identifier for health and social care use. Researchers were blind to the participants. Data were collected for 12 months before and after the intervention to minimise the effects of seasonality (as this was a new service).

### Study size

The study size calculation was based on the effect seen in the Cornwall Pathfinder SP pilot (23% increase WEMWBS) (Cell Consulting Ltd, 2015; Murray, 2016), and a standard deviation of 8.97 (12.5%) drawn from population studies (Stewart-Brown and Janmohamed, 2008), as this was not provided in current studies. Powered at 80% and a 5% significance level, the study would require 170 people to be statistically conclusive, roughly equivalent to the first six months of participants (1 July–31 December 2016)

### Statistical methods

The health outcomes and health and social care service use and cost were analysed by mean change in outcome or service contacts or costs 12 months before and after the first point of contact with the link-worker. For metric and interval outcomes (WEMWBS, Well-being Star and PAM), a paired *t*-test was used to test for significance, if the Shapiro–Wilk test for normality was not significant (Ghasemi and Zahediasl, 2012). Non-normally distributed metrics and interval outcomes and ordinal outcome measures (age band, PAM level, RCFS and Goals) were tested using the Wilcoxon matched-pair signed-rank test. Categorical outcomes such as referral route (acute and community) were analysed using the two-sample *t*-test or the Mann–Whitney test, if either analytic arm was identified as not normally distributed. Where more than two categories were analysed, the X^2^ test was used. For bivariate linear regression, the Pearson *r* correlation test was used, where both metric variables were normally distributed. Ordinal correlations used the Spearman’s rank correlation.

To analyse the impact of age, referral route, PAM level and RCFS score on entry on health and social care costs, a multiple regression model was developed. Each variable tested for statistical significance before inclusion in the model, until the best fit was determined. Due to the small numbers of individuals with missing variables (age *n* = 1, PAM Level: *n* = 5) or without 12-month post-intervention, health service data were not imputed for that variable or used in the modelling. All analyses were undertaken in SPSSv24.

### Consent and ethical approval

Service users were consented for the use of their health outcomes and primary and secondary care service activity data in a service evaluation. The evaluation was supported by university-employed Researchers-in-Residence holding honorary positions with Torbay and South Devon NHS Foundation Trust, operating under ethics granted by the NHS Health Research Authority (Research Ethics Committee reference: 17/LO/1745; Protocol number: PSMD-208147-SA-FG-034; Integrated Research Application System project ID: 208147).

## Findings

### Participants and characteristics

**Figure 2.**
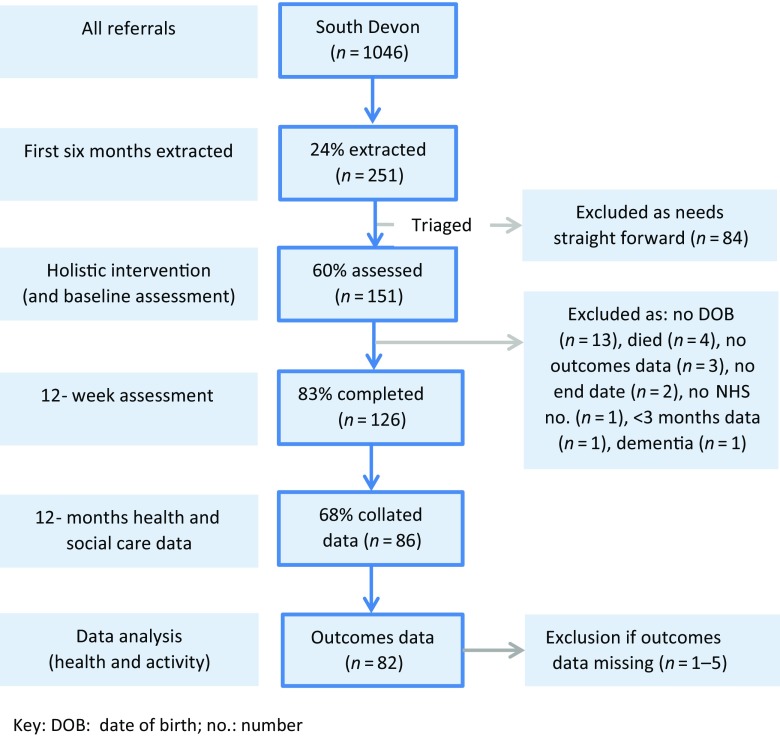
Flow diagram of study participants. Key: DOB = date of birth; no. = number

There were 1046 participants; 251 were referred in the first six months, 151 (60%) were triaged to receive a longer, guided conversation and the 12-week programme. Figure 2 shows the referral route and numbers entering the ‘holistic’ service in the first six months. There was a wide variation in triage rates by voluntary sector providers (12–72%).

A total of 126 participants had health outcomes data. However, only 86 (68%) had a full 12 months’ worth of health and social care data after the first point of contact. Outcome data were missing on 5 variables in 13 individuals: age (1), PAM score (5), Well-being Star (4) and RCFS score (4) and referral source (1).

Table 1 shows that the age distribution was negatively skewed towards the elderly, with a mean of 79.6 years (median of 82.2 years with range 52.7–94.5), older than in other studies (Grant *et al*., 2000, Grayer *et al*., 2008; Friedli *et al*., 2012; Dayson and Bashir, 2014; Bertotti *et al*., 2015; Farenden *et al*., 2015; Kimberlee, 2016; Windle *et al*., 2016). Participants were lowly activated with nearly three-quarters with PAM scores at Levels 1 and 2, 11% higher than population estimates in over 65-year-olds with one or more long-term conditions (PAM Level 1, 16% and PAM Level 2, 45%) (Blakemore *et al*., 2016). The RCFS scores varied between 1 and 9. This was not normality distributed, with a median and mode of 4, with most people falling into the categories of vulnerable, capable of limited activities but not dependent on others (4) to moderately frail (6), requiring help with outside activities and housekeeping. In relation to health and social care use, most distributions were positively skewed, with 45 (52.3%) participants having had no A&E contacts in the year prior to the intervention, 48 (55.8%) no in-patient care, 22 (25.6%) no outpatient care, 25 (29.1%) no community care and 75 (87.2%) no social care contact. Multiple use of different types of health and social care services was more normally distributed, with a mean of 2.5, varying from 10 participants with no contact to nearly a third of participants (30.3%) contacting 4 or 5 kinds of services, which together represent a disproportionate 2624 contacts (66.7% of the total) in the year before entry into the programme.

**Table 1.** Participant demographics and entry-level outcomes variables

Variable	No. of people (%)	Variable	No. of people (%)
Age band in years (*n* = 85)		PAM (*n* = 81)	
50–59	5 (5.8)	Score Level	
60–69	10 (11.6)	Level 1	42 (51.9%)
70–79	23 (26.7)	Level 2	19 (23.5%)
80–89	38 (44.2)	Level 3	14 (17.3%)
90+	9 (10.4)	Level 4	6 (7.4)
Sex (*n* = 86)		Referral type (*n* = 85)	
Female	63 (73.3)	Community	48 (55.8)
Male	23 (26.7)	Complex	37 (43.0)
Referral route (*n* = 86)		HSC use (≥1) (*n* = 86)	
GP	21 (24.4)	A&E/MIU	41 (47.7)
Hospital discharge	7 (8.1)	In-patient	38 (44.2)
MDT	28 (32.6)	Outpatient	64 (74.4)
MDT SC	4 (4.7)	Community	61 (70.9)
Other statutory	2 (2.3)	Social care	11 (12.8)
Self-referral	6 (7.0)	Multiple HSC use (*n* = 86)	
Voluntary sector	17 (19.8)	0	10 (11.6)
RCFS score (*n* = 83)		1	13 (15.1)
Score ≤3	7 (8.4)	2	19 (22.1)
Score 4	36 (43.3)	3	18 (20.9)
Score 5	11 (13.3)	4	20 (23.3)
Score ≥6	29 (35.0)	5	6 (7.0)

PAM = Patient Activation Measure; HSC = Health and Social Care; GP = General practice; A&E = Accident and Emergency; MIU = Minor Injury Units; MDT = multidisciplinary team; MDT SC = MDT social care; RCFS = Rockwood Clinical Frailty Scale.

### Health outcomes

Table 2 shows the mean change in health outcomes on entry and at 12 weeks or exit. Goal achievement and all health and social outcomes showed statistically significant change. The average number of goals per participant was 3.2 (median 3; range: 1–7), with most people (85.3%) achieving their goals in 12 weeks. Participants’ average WEMWBS score on entry was 38.8, compared to a population mean of 51 (Stewart-Brown and Janmohamed, 2008), with 13 people with scores under 30 (very low). WEMWBS scores showed a mean increase of 20.3% (*P* = 0.000) (Stewart-Brown and Janmohamed, 2008). A meaningful change (≥5 points) was seen in 54 people (62.9%). Well-being Star showed an average improvement of 43.3% (*P* = 0.000). There was a 22.8% increase in PAM score, which equated to 0.7 of a PAM Level. Over half of users saw their PAM Level increase by 1 or more (45/81, 55.6%).

**Table 2.** Mean change in health and social care outcomes

Variable	N	Mean score on entry (SD) (95% CI)	Mean score at 12 weeks (SD) (95% CI)	Change in mean score (%) (95% CI)	*P*-value
WEMWBS	86	38.8 (10.3) (36.6–41.0)	46.7 (10.9) (44.4–49.1)	7.9 (20.3%) (6.1–9.7)	0.000^a^
Well-being Star™	82	30.6 (12.6) (27.9–33.4)	43.9 (13.3) (41.0–46.8)	13.3 (43.3%) (10.6–15.9)	0.000^a^
PAM					
Score	81	50.1 (11.7) (47.5–52.7)	61.6 (16.9) (57.8–65.3)	11.4 (22.8%) (8.6–14.3)	0.000^b^
Level	81	1.8 (1.0)	2.6 (1.1)	0.7 (38.9%)	0.000^b^
RCFS	82	4.8 (1.3)	4.5 (1.6)	0.2 (4.7%)	0.033^b^
Goal achievement	85	3.2 (1.3)	0.5 (0.7)	2.7 (85.3%)	0.000^b^

SD = Standard Deviation; 95% CI = 95% Confidence Interval; WEMWBS = Warwick-Edinburgh Mental Well-being Scale; PAM = Patient Activation Measure; RCFS = Rockwood Clinical Frailty Scale.

a Student’s Paired *t*-test.

b Wilcoxon Matched-Paired Signed-Rank Sum test.

The RCFS showed a decrease of 4.6% towards less frailty. A decrease in one or more levels was seen in 28 people (34.1%), 46 (56.1%) saw no change and 8 people (9.8%) saw an increase in frailty, 4 people by 3 levels.

### Health and social care use

The mean change in health and social care use is shown in Table 3. GP contact data were not included due to the poor quality of extracted data. The increase in mean health and social care use for all service types was only statistically significant for in-patient, community and social care. Most distributions were highly positively skewed towards 0, 1 or 2 contacts. A relatively small number of participants (10, 11.6%) accounted for a large proportion of the increase across the health and social care systems (79.4%). In social care, 64 (73.5%) people saw a decrease or no change in use whilst in health care it was 32 (37.2%).

**Table 3.** Mean change in health and social care use (*n* = 86)

Variable	Average contacts 12 months before entry (range)	Average contacts 12 months after entry (range)	Change in mean score (%)	*P*-value^a^
A&E	0.99 (0–7)	1.33 (0–6)	0.34 (34.0%)	0.137
In-patient	1.19 (0–9)	1.99 (0–15)	0.80 (67.4%)	0.018
Outpatient	4.06 (0–31)	4.52 (0–35)	0.46 (11.5%)	0.560
Community	9.28 (0–81)	16.24 (0–96)	6.97 (75.1%)	0.001
Social care	30.20 (0–730)	113.04 (0–1290)	82.84 (274.3%)	0.000

A&E = Accident and Emergency.

a Wilcoxon Matched-Paired Signed-Rank Sum test.

### Health and social care costs

Table 4 shows the sum and mean change in health and social care costs and the relative cost contributions.

**Table 4.** Sum and mean change in health and social care costs 12 months before and after entry into the service^a^

Variable	Sum of costs 12 months before	Sum of costs 12 months after	Change in costs	Average cost 12 months before	Average cost 12 months after	Change in mean costs	% change	*P*-value^b^
Health costs	£330 360	£547 510	£217 150	£3841	£6366	£2525	66.7%	0.004^b^
Social care costs	£57 123	£202 196	£145 073	£664	£2351	£1686	254.0%	0.000^b^
Total cost	£387 483	£749 706	£362 223	£4506	£8718	£4212	93.5%	0.001^b^
% change	–	–	–	85.3%	73.0%	59.9%	–	–

a Costs exclude GP contacts.

b Using the Wilcoxon Matched-Paired Signed-Rank Sum test.

The total sum of health and social care cost before and after was £387 483 and £749 706, respectively. Thus, there was an overall increase in costs of £362 223 (93.5%). This represented a change in mean cost of £4212 per user, a statistically significant increase in health and social care. Although health care costs accounted for the majority of costs (85.3%) and the overall cost increase (59.9%), social care costs also increased, by more than twofold.

Just over half of users (55.8%, 48/86) saw an increase in costs (>£400), 15 (17.4%) saw no change (−£400 to £400) and 23 (26.7%) saw a reduction in costs (<−£400). Of those with an increased cost, 28 (29.2%) saw an increase >£5000 per person.

To further understand the large increase in cost, we examined the records of 13 participants in 1 locality with costs >£5000 (out of 28 overall), who accounted for 59.1% of the overall cost increase for all localities (£213 960 of £362 223). Ten were referred by intermediate care (three re-referrals) and three by the voluntary sector. The cases revealed that health costs accounted for 81.2% of the overall increase cost. The 10-fold increase largely resulted from hospitalisation (with an average change of 36 days) and increased contact with community services, due to an escalation in morbidity and frailty (and mental health for many), caused by degenerative or rare diseases, long-term conditions and discrete episodes like falls, urinary tract infections, sepsis and end-of-life care (4). Almost all the social care component was due to one young person who died unexpectedly from a rare condition, incurring a large social care cost (>£30 000). Case notes revealed that the Well-being Co-ordinators were acting as key workers in many cases, improving continuity of care, reducing social isolation, providing mental health and carer support during a difficult time.

### Influence of participants’ entry-level variables on health and social outcomes

We explored the association between age bands (<75, 76–85, >85), sex, PAM Level (1–4) and RCFS score (1–9) on entry to the programme and meaningful change in WEMWBS score (≥5) and Well-being Star™ scores, in order to identify subgroups who may benefit from the service more than others.

Table 5 shows the proportion of users achieving meaningful change in WEMWBS (defined as ≥5 point change (Putz *et al*., 2012)) across variables. Over 60% of participants showed meaningful change in WEMWBS by variable, with minor variation within category. For ordinal variables (age, PAM Level and RCFS score), the proportion of people with meaningful change was fairly consistent across subgroups, and any variation seen was not statistically significant. There were similar results for nominal variables (sex and referral route), with subgroup variation not statistically significant.

**Table 5.** Association between meaningful change in health outcome and entry-level variables

Variable	Number (*n*)	Meaningful change in WEMWBS score (≥5)		% with change (≥5)	*P*-value
		No	Yes		
Sex	*n* = 86	32	54	62.8%	0.432^a^
Female	63	25	38	60.3%	
Male	23	7	16	69.6%	
Age	*n* = 85	32	53	62.4%	0.928^a^
≤75	26	9	17	65.4%	
76–85	28	11	17	60.7%	
>85	31	12	19	61.3%	
PAM Level	*n* = 81	29	52	64.2%	0.547^b^
Level 1	42	15	27	64.3%	
Level 2	19	9	10	52.6%	
Level 3	14	4	10	71.4%	
Level 4	6	1	5	83.3%	
RCFS	*n* = 82	30	52	62.7%	0.752^b^
≤3	7	2	5	71.4%	
4	35	11	24	58.6%	
5	11	5	6	54.5%	
≥6	29	12	17	69.4%	
Referral route	*n* = 85	32	53	62.4%	0.371^b^
GP	21	10	11	52.4%	
Hospital discharge	7	2	5	71.4%	
MDT	29	10	19	64.3%	
Other statutory	3	0	3	100.0%	
Staff referral	6	3	3	50.0%	
Social care	2	2	0	70.6%	
Voluntary sector	17	5	12	50.0%	
Referral route (grouped)	*n* = 85	32	53	62.4%	0.499^a^
Community	48	20	28	58.3%	
Complex	37	12	25	67.6%	

WEMWBS = Warwick-Edinburgh Mental Well-being Scale; PAM = Patient Activation Measure; RCFS = Rockwood Clinical Frailty Scale; GP = General Practitioner; MDT = MultiDisciplinary Team meeting; Other statutory, for example, public health services.

a *X*^2^ test.

b Fisher’s exact test.

### Predicting change in health and social outcomes, activity and cost with entry-level variables

As the data were not normally distributed, binominal logistic regression models were developed to explore the influence of entry-level variables, age, sex, PAM and RCFS score on health care, social care and health and social care costs (<£400 and ≥£400). Only the change in social care cost regression model was statistically significant, with the RCFS score only explaining 24.6% of the variance (Nagelkerke R-squared test) and correctly classifying 82.1% of cases. Increasing RCSF is likely to predict change in social care costs ≥£400 by 2.49 times (95% CI: 1.43–4.33, *P* = 0.001).

## Discussion

This before-and-after study evaluated the impact of a 12-week holistic link-worker intervention on people over 50 years old with multiple long-term conditions, as part of a SP programme in South Devon. Its aims were to reduce social isolation, improve health and activation, and to reduce the use of health and social care services. Our cohort was 73% female, elderly (nearly half older than 80 years) and frail with nearly half vulnerable, unable to manage many activities of daily living. The majority had been referred from intermediate care, general practice or the voluntary sector and were lowly activated to manage their health. Most participants were users of outpatient or community services, with just over a quarter of all participants in contact with all health and social care service types (higher suers), accounting for two-thirds of all contacts.

Most users achieved all their living-well goals set with Co-ordinators and showed a statistically significant, meaningful change in their health and social care outcomes over the following 12 weeks. The largest mean change was in the Outcomes Star™, which given the magnitude is likely to have increased scores on most of its seven dimensions (such as looking after yourself, social participation and feeling positive). This was corroborated by an increase in WEMWBS, indicating improvements in people’s functioning, social relationships, sense of purpose as well as feelings of well-being and happiness (Fat *et al.*, 2017).

Over half of users saw an increase in their PAM level by 1 or more levels, suggesting that participants felt more able to manage their health condition, and a third saw improvement in frailty level by 1 or more. These changes were supported by many qualitative case studies that documented significant changes to peoples’ lives socially, physically and mentally, brought about by working with Co-ordinators to address their social, physical and economic needs.

However, these positive changes were not accompanied by a uniform decrease in health and social care use. Although just under half of the cohort saw a decrease or no change in activity, on average there was an overall increase in activity and costs, significant for in-patient, community and social care services. A significant proportion of this increase was accounted for by a rapid, escalation in morbidity and frailty in just over a dozen people. Given that most of this elderly cohort were referred by intermediate care, a rapid deterioration in health might not be unexpected, and not necessarily preventable by a predominantly biopsychosocial intervention (Parkinson and Buttrick, 2015).

Our primary findings emphasise the importance of better understanding the types of people who would benefit most from SP. Our analysis of different categories of entry-level variables showed some variation in health outcomes and service use, but with no discernible patterns or only weak, non-statistically significant associations. The exception was the RCFS score, which was positively correlated with change in social care costs, possibly explained by people becoming less mobile and able to manage the activities of daily living with time and, therefore, becoming more dependent on social support. This implies that participants reporting a reduction in their frailty, through being supported to be more socially active and engaged, are associated with a relatively small increase in social care costs. In practice, our results suggest that the majority of older people over 50 with long-term conditions stand to benefit from holistic SP irrespective of age, sex and levels of activation and frailty, including the frail elderly.

### Strengths and limitations of study

This study was conceptually clearer than previous studies, with a specific focus on referrals receiving the holistic link-worker approach (rather than all referrals irrespective of input intensity). We included relevant outcomes not included in other studies – patient activation and frailty – that relate to assumed benefits of SP. We had good participation and excellent follow-up rates in relation to outcomes and long-term follow-up of health and social care activity data and costs. This enabled us to assess the impact on health and social care use with greater certainty, with less concern for regression to the mean effects (Steventon *et al.*, 2011; Murray, 2016) or the influence of seasonality (a potential issue when the first studied cohort service commenced in summer). Few studies have collected longer-term data (Brandling *et al.*, 2011; Dayson and Bashir, 2014; Kimberlee *et al.*, 2014; Maughan *et al.*, 2016) with such high-level follow-up (Grayer *et al.*, 2008) or such a comprehensive range of acute, community and social care activity and cost data (Bickerdike *et al.*, 2017). This allowed some provisional exploration of which patient characteristics on programme entry might be associated with positive outcomes. Although our numbers were small in some subcategories (which may have underpowered this element of the analysis), we are not aware of other studies that have done this.

The lack of a control group means that we can only assume a tentative causal link between the intervention and the findings. Other studies have found improved outcomes and reduced activity in control groups (Bertotti *et al.*, 2015; Murray, 2016), suggesting that the findings could be biased positively. Conversely, in a relatively frail, elderly cohort where an increase in health and social care activity is more likely, a control group could have helped determine how much was prevented. The large variation in triage rates might suggest different criteria were being used in practice. There was no outcome or attendance data for activities collected at 12 months, which would have strengthened causal claims between the intervention and change in health and social care use. Data quality issues precluded an analysis of GP contacts data. Local costings were used (based on a national methodology) rather than unit cost data (Curtis and Burns, 2016) more typically used in economic evaluations. However, we present the costs used in our calculations so as to facilitate comparison with other studies. We did not include the average cost per person of the holistic element of the Well-being Coordination service (which cost £165 500 and managed 1046 referrals in the first year) as contact time was not recorded, nor the wider societal benefits of the programme (eg, increased employment, access to benefits or volunteering) required to estimate Social Return On Investment (Dayson and Bashir, 2014).

### Comparison with existing literature

The magnitude and direction of improvement in health and well-being outcomes is broadly supportive of other studies (Murray, 2016), including those with stronger designs, but few studies have used WEMWBS (Murray, 2016) or Outcomes Star, and lack of sufficient detail to allow direct comparisons (Friedli *et al.*, 2012).

SP is often targeted at all ages, often in more deprived communities. This programme is unusual in focusing on people over 50 years old with two or more long-term conditions. We are only aware of a few evaluations that report similar median ages to our study (Dayson and Bashir, 2014; Windle *et al.*, 2016), but these have not captured the impact on frailty. Over 50% of our cohort was mildly or moderately frail, many referred by their GP or the MDT. Our study indicates a potentially small but significant impact, shifting people from mildly frail to vulnerable, or dependency on others for their activities of daily living to greater confidence, mobility and independence.

Frailty is not a static classification; it can become better or worse (British Geriatrics Society, 2014). Evidence suggests that addressing mobility, social isolation and loneliness, physical activity and low mood, through interventions like SP, can reduce severity and improve outcomes (British Geriatrics Society, 2014). Ensuring integrated community and primary care to support people with frailty is advocated nationally (Baker *et al.*, 2016). This study supports the emerging evidence base on innovations in this area and suggests that reversing frailty could have positive consequences for the cost of social care.

### Implications for research and practice

There are only two studies (of which we are aware) that have assessed the impact of SP on patient activation (using PAM) (Mendip, 2016; Bertotti *et al.*, 2017). Like our study, these showed positive improvements in participants’ levels of activation to manage their long-term health condition, many from a low level, but also those at higher activation levels. However, our study had high levels of follow-up, reducing the risk of a positive bias, and was statistically significant for score and level. The smaller effects seen in Bertotti *et al.’s* (2017) study might be explained by the use of ‘light-touch’ rather than ‘holistic’ link-workers or differences in the referred cohort. People who are less activated are less receptive to preventative advice and care, less active in decisions about their care, and adhere less to treatment regimens (Hibbard *et al.*, 2013). As we did not review clinical indicators, our findings only partially support the assumption in national policy that SP improves patients’ self-management. PAM is also of interest to national policy-makers because it can be used as a predictor of current and future health and social care costs (Hibbard *et al.*, 2013). However, our study did not find such an association.

Evidence on SP reducing health and social care activity is generally favourable (Polley *et al.*, 2017a), but only two studies had a control group (Bertotti *et al.*, 2015; Murray, 2016), with follow-up at 4 and 12 months. In contrast to this study, evaluations show reductions in A&E attendance (8–26.8%), emergency admissions (6–33.6%) and secondary care referrals (55%). This did not appear to be the case in our study, with a small cohort of people, mostly referred from intermediate care (rather than general practice), subsequently experiencing a rapid deterioration in health, accounting for much of the increase. As other studies analysed all link-worker referrals together and had low rates of follow-up (Dayson and Bashir, 2014), it is possible that these results were positively biased, with those people having a positive experience of SP being more likely to be followed up. The study with the least risk of bias (a randomised controlled study) saw a doubling of referrals to mental health service (Grant *et al.*, 2000). Larger controlled studies with good follow-up are now required to assess the impact of a link-worker on the frail, elderly, in order to determine whether policy in this area should be developed.

## Conclusion

This study focused on the impact of ‘holistic’ link-working, as part of a wider SP service mainly offering ‘lighter touch’ link-working – the approach typically reported in UK studies. It showed improved quality of life, patient activation and reduced frailty in a complex cohort with multiple long-term conditions. Just under half of referrals saw a decrease or no change in cost and activity after 12 months. For those with an increase, this was primarily due to a small group of people experiencing a rapid deterioration in health. Further, controlled studies are needed to strengthen claims of causality.
